# A jump in the atrioventricular conduction curve is not caused by a switch from fast pathway to slow pathway conduction

**DOI:** 10.3389/fphys.2024.1367509

**Published:** 2024-03-29

**Authors:** Youhua Zhang

**Affiliations:** ^1^ Departments of Cardiovascular Medicine and Molecular Cardiology, The Cleveland Clinic, Cleveland, OH, United States; ^2^ Department of Biomedical Sciences, New York Institute of Technology College of Osteopathic Medicine, Old Westbury, NY, United States

**Keywords:** atrioventricular node, discontinuous conduction curve, dual-pathway electrophysiology, intranodal reentry, nodal–atrial reentry, His electrogram alternans

## Abstract

**Background:** A jump in the atrioventricular (AV) conduction curve is the current clinical criterion of dual-pathway electrophysiology. However, the assumption that a jump indicates a switch from fast pathway (FP) to slow pathway (SP) conduction remains unconfirmed. This study was carried out to investigate whether a jump indeed indicates a transition from FP to SP conduction, and if not, what the potential cause is.

**Methods:** Eighty-one experimental records from rabbit AV nodal preparations containing the following data were analyzed: 1) had at least one AV conduction curve and 2) had recording of His electrogram alternans (a validated new index of dual-pathway conduction). Most cases also had intracellular action potential recordings from the AV nodal fibers.

**Results:** Of the 81 preparations, 11 (13%) showed a jump in the AV conduction curve. The jumps always occurred after the FP to SP transition. The FP–SP transition occurred at prematurity at 196 ± 39 ms *versus* the jump at 114 ± 13 ms (*p* < 0.001). The beat with a jump showed an SP–FP pattern in seven and an SP–SP pattern in four preparations. The jumps were always associated with and most likely caused by the formation of intranodal/nodal–atrial reentry and its subsequent conduction, rather than a switch from FP to SP conduction.

**Conclusion:** Contrary to what has been assumed, a transition from FP to SP conduction does not produce a jump in the AV conduction curve. A jump in the AV conduction curve is most likely caused by the formation of intranodal/nodal–atrial reentry and its subsequent conduction.

## Introduction

The dual-pathway atrioventricular (AV) nodal electrophysiology is the underlying basis of AV nodal reentrant tachycardia (AVNRT), the most common type of paroxysmal supraventricular tachycardia (PSVT) (Scheinman and Yang, 2005; Gonzalez et al., 2020). A “jump” in the AV conduction curve (aka a discontinuous curve) remains the current clinical criterion for diagnosing dual-pathway electrophysiology (Zhang, 2016; Gonzalez et al., 2020). It has been assumed and accepted that the fast pathway (FP), which has a longer effective refractory period (ERP) and produces a shorter AV delay, is responsible for AV conduction at slow heart rates (or longer cycle length). On the other hand, the slow pathway (SP) has a shorter ERP but generates a much longer AV delay and is responsible for AV conduction at fast heart rates (or shorter cycle length). Thus, a switch from FP to SP conduction would result in a significant increase in AV conduction time, commonly known as a jump in the AV conduction curve (Zhang, 2016; Gonzalez et al., 2020); however, this has never been confirmed.

Because dual-pathway electrophysiology is the underlying cause of AVNRT, the dual pathways were initially believed as pathology, which are found only in patients with AVNRT. However, it has been known that dual pathways can be found in most patients, even in those without AVNRT (Denes et al., 1975; Zhang, 2016; Gonzalez et al., 2020). In addition, it is also recognized that AVNRT patients could display a smooth AV conduction curve (Sheahan et al., 1996). Hence, a smooth curve does not rule out the existence of dual pathways (Zhang, 2016; Gonzalez et al., 2020). Over the years, we have discovered and validated a novel index of dual-pathway conduction. It was initially termed His electrogram (HE) alternans (Zhang et al., 2001; Zhang et al., 2003; Zhang et al., 2004; Zhang and Mazgalev, 2011; Zhang and Mazgalev, 2012; Zhang, 2014). With this new index, we have demonstrated that dual pathways are ubiquitous normal electrophysiology, existing in all subjects. Now, the inevitable question is why only some patients have a jump in their AV conduction curve if all subjects have dual pathways.

By using HE alternans as a novel index of dual-pathway conduction, we have investigated in this study whether a jump in the AV conduction curve is indeed caused by the transition from FP to SP conduction, and if not, what the potential cause for such a jump could be.

## Materials and methods

This study was conducted by analyzing our previous experimental records obtained from rabbit AV nodal preparations since our discovery and validation of HE alternans as a novel index of dual-pathway conduction, first reported in 2001 (Zhang et al., 2001). All experiments contained the following: 1) data collected during programmed (A1A2 or A1A2A3) atrial pacing and an AV nodal conduction curve that could be generated and 2) recordings of HE alternans. Most cases also had intracellular action potential (AP) recordings from the AV nodal fibers. Experiments from 81 consecutively collected AV nodal preparations were included in the analysis. Most of the experiments were conducted at Cleveland Clinic during Dr. Zhang’s employment there. Although specific data generated from these same rabbit preparations had been used in our previous publications (Zhang et al., 2001; Zhang et al., 2003; Zhang et al., 2004; Zhang and Mazgalev, 2011; Zhang and Mazgalev, 2012; Zhang, 2014), the analyzed results in this report are new and have not been previously published. The use of animals was approved by the Institutional Animal Care and Use Committee and follows the “Guide for the Care and Use of Laboratory Animals” (NIH Publication No. 85-23, Revised 1996).

### *In vitro* rabbit AV node preparations

The experiments were conducted on atrial–AV node preparations from adult New Zealand white rabbits of both sexes, as previously described (Zhang et al., 2001; Zhang et al., 2003; Zhang et al., 2004; Zhang and Mazgalev, 2011; Zhang and Mazgalev, 2012; Zhang, 2014). In brief, after anesthesia was given using sodium pentobarbital (50 mg/kg) and opening of the chest, the heart was removed and placed in standard oxygenated Tyrode’s solution saturated with 95% O_2_/5% CO_2_ at a flow rate of 35 mL/min. After trimming, the AV node preparation contained the triangle of Koch and the surrounding right atrial and ventricular tissues.

### Electrical recordings and stimulation protocols

As described previously (Zhang et al., 2001; Zhang et al., 2003; Zhang et al., 2004; Zhang and Mazgalev, 2011; Zhang and Mazgalev, 2012; Zhang, 2014), custom-made bipolar electrodes (0.2-mm spacing) were used to record atrial electrograms and for atrial pacing. Roving bipolar electrodes were used to record the superior and inferior His electrograms. All electrodes were positioned with micromanipulators (M330, WPI, Sarasota, FL). An eight-channel, programmable stimulator (Master-8, AMPI, Jerusalem, Israel) was used for pacing. The electrical signals were amplified, filtered at 30–3,000 Hz (CyberAmp 380, Axon Instruments, Union City, CA), recorded, and analyzed using AxoScope (Axon Instruments).

Intracellular APs from AV nodal fibers were recorded using standard glass microelectrodes. The anatomical location, AP morphology, amplitude, and dV/dt (∼10–50 V/s), as well as cycle-length dependency, were used to identify signals originating from different nodal regions. As reported previously (Zhang, 2014), the AP from fibers in the superior nodal domain typically shows two wave fronts at a short premature beat: the first is caused by the antegrade FP wave front and the second is caused by the “retrograde” SP wave front. The AP from fibers in the inferior nodal domain usually does not show two wave fronts.

A programmed atrial pacing protocol (A1A2) was used in a majority of preparations, with a basic cycle length (A1A1) of 300 ms. An A1A2A3 pacing protocol was utilized only in a dozen preparations from which multiple conduction curves were available. A standard AV nodal conduction curve was generated by interposing a premature stimulus A2 (or A3) after every 20th basic beat A1. The premature coupling interval A1A2 (or A2A3) was progressively shortened (in steps of 10–5 ms) until an AV block occurred. The AV nodal ERP was defined as the longest A1A2 that failed conduction by the AV node.

### Monitoring AV nodal fast and slow pathway conduction using His electrogram alternans

HE alternans was used to monitor FP and SP conduction, as previously reported (Zhang et al., 2001; Zhang et al., 2003; Zhang et al., 2004; Zhang and Mazgalev, 2011; Zhang and Mazgalev, 2012; Zhang, 2014). The HE recorded from the superior His bundle domain (superior His electrogram, SHE) is high in amplitude during FP conduction, and the amplitude becomes low during SP conduction. In contrast, the HE recorded from the inferior His bundle domain (inferior His electrogram, IHE) is always from low amplitude during FP to high amplitude during SP conduction. In addition to the amplitude changes, timing changes also occur between the corresponding SHE and IHE during dual-pathway conduction. During FP conduction, the SHE leads the corresponding IHE. The sequence is reversed during SP conduction.

### Definition of the jump in the AV conduction curve

A jump in the AV conduction curve was defined as an increase of 50 ms or more in the atrial-His (AH) interval when atrial prematurity (A1A2) was shortened by 10 ms, which is the same as the clinical criterion used in patients (Zhang, 2016; Gonzalez et al., 2020).

### Statistical analysis

Data are presented as the mean ± SD where appropriate. A comparison between the prematurity resulting in a transition from FP to SP conduction and the prematurity resulting in a jump was performed using Student’s *t*-test. *p* < 0.05 was considered statistically significant.

## Results

### FP to SP transition and the jump in the AV conduction curve

**FIGURE 1:**
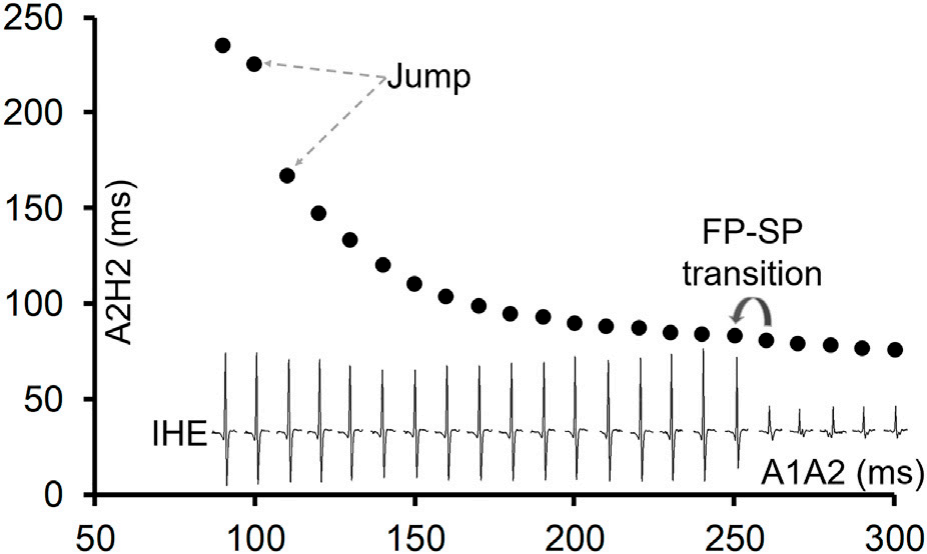
An example of the AV conduction curve with a jump and His electrogram (HE) alternans. The AV conduction curve was plotted by using the atrial premature coupling interval (A1A2) in the abscissa and the atrial His interval (A2H2), representing the AV conduction time, in the ordinate. A jump was observed at A1A2 = 100 ms, when the A2H2 increased from 167 ms at A1A2 = 110 ms to 225 ms. The inferior His electrogram (IHE) corresponding to each A1A2 was plotted under the curve. Judging by the HE alternans, the transition from the fast pathway (FP) to slow pathway (SP) conduction occurred at A1A2 = 250 ms (where the IHE amplitude suddenly increased). However, the FP to SP transition was smooth.

Of the 81 preparations, 11 (13%) showed a jump in the AV conduction curve. An example is shown in Figure 1. In all preparations, the transition from FP to SP conduction was relatively smooth (without a jump). The jump always occurred after the FP to SP transition at a much shorter prematurity near the end of the curve at the short A1A2 region or the left side of the curve (Figure 1). On average, the FP to SP transition occurred at prematurity at 196 ± 39 ms *versus* the jump at 114 ± 13 ms (*p* < 0.001), whereas the AV nodal ERP was 93 ± 12 ms.

### The beats with a jump and dual-pathway conduction

**FIGURE 2:**
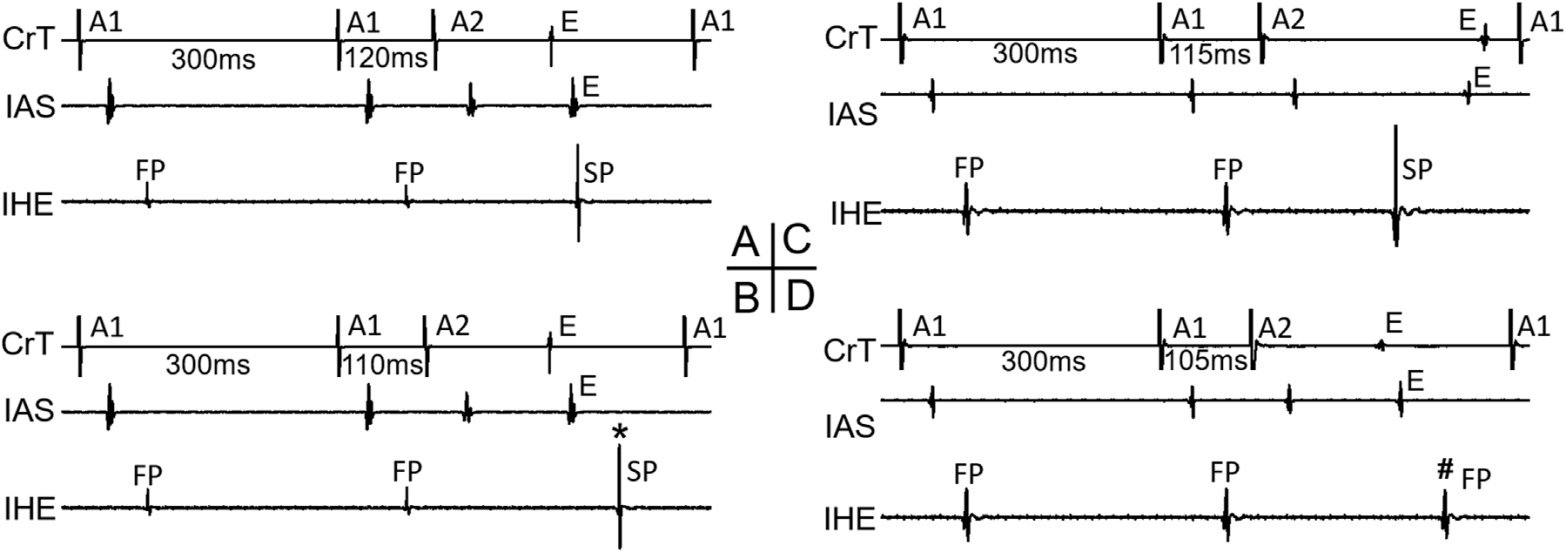
Beats with a jump and His electrogram alternans. Examples of a beat with a jump that resulted in either an SP pattern [**(A,B)**, *] or an FP pattern [**(C,D)**, #] of His bundle activation are shown. Atrial electrograms from the interatrial septum (IAS) and crista terminalis (CrT), together with the inferior His electrogram (IHE), recording at the premature beat preceding the jump **(A,C)** and with the jump **(B,D)** are shown, respectively. **E** atrial echo beat induced by the premature beat.

As all jumps occurred after the FP-to-SP transition, the beat preceding the jump was always conducted by the SP. At the beat resulting in a jump, the HE alternans showed an SP–SP pattern in 4 of the 11 preparations (Figures 2A, B) and an SP–FP pattern in 7 of the 11 preparations (Figures 2C, D). Note that here we used “FP pattern or SP pattern” instead of FP conduction or SP conduction to indicate the fact that the beat resulting in a jump most likely failed to directly conduct to the His bundle; instead, the premature beat induced intranodal or nodal–atrial reentry and the subsequent reentrant beat conducted to the His bundle, as will be explained later.

### Evidence of intranodal/nodal–atrial reentry formation at short prematurities after the FP to SP transition

**FIGURE 3:**
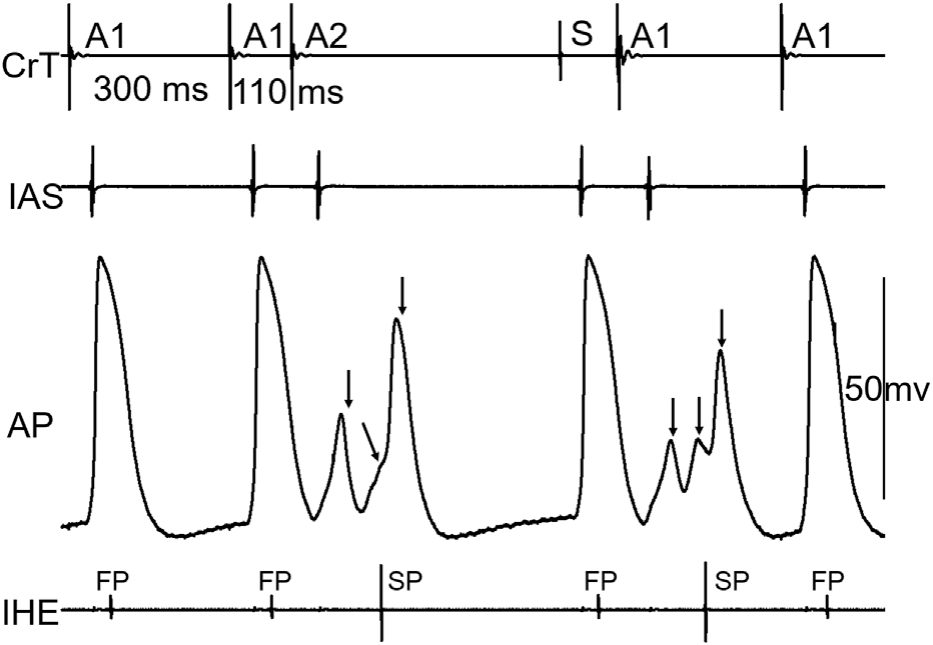
Example of concealed intranodal reentry. Action potential (AP) recording from a nodal fiber revealed multiple wave fronts (arrows) induced by the premature beat, indicating the formation of complex intranodal reentry. However, these events were not reflected on neither the atrial nor His electrogram. IAS, interatrial septum; CrT, crista terminalis; IHE, inferior His electrogram; S, sinus beat. Note that the A1 beat immediately following the sinus beat (S) acted as a premature beat.

Figure 3 shows an example that at a short atrial premature beat (A1A2 = 110 ms), complex multiple wave fronts (indicating complex intranodal reentry) could be detected by the AP recording from a fiber in the center of the node. These reentrant wave fronts were not associated with any atrial echo beat or extra His bundle activation, indicating concealed intranodal reentry.

**FIGURE 4:**
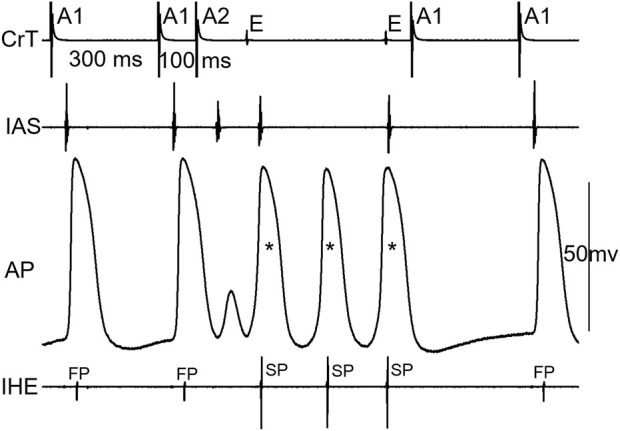
Example of intranodal tachycardia revealed by AP recording. Note that there were 3 nodal activations (*) versus 2 atrial echo beats (not 1:1 nodal-atrial activation). IAS, interatrial septum; CrT, crista terminalis; IHE, inferior His electrogram; E, atrial echo beat.

Figure 4 shows an example that a short atrial premature beat (A1A2 = 100 ms) initiated intranodal tachycardia. The intranodal tachycardia could be detected by the AP recording. Note that the intranodal reentrant beats were only partially conducted to the atria, indicating that the atrial tissue was not a critical component of this intranodal tachycardia.

**FIGURE 5:**
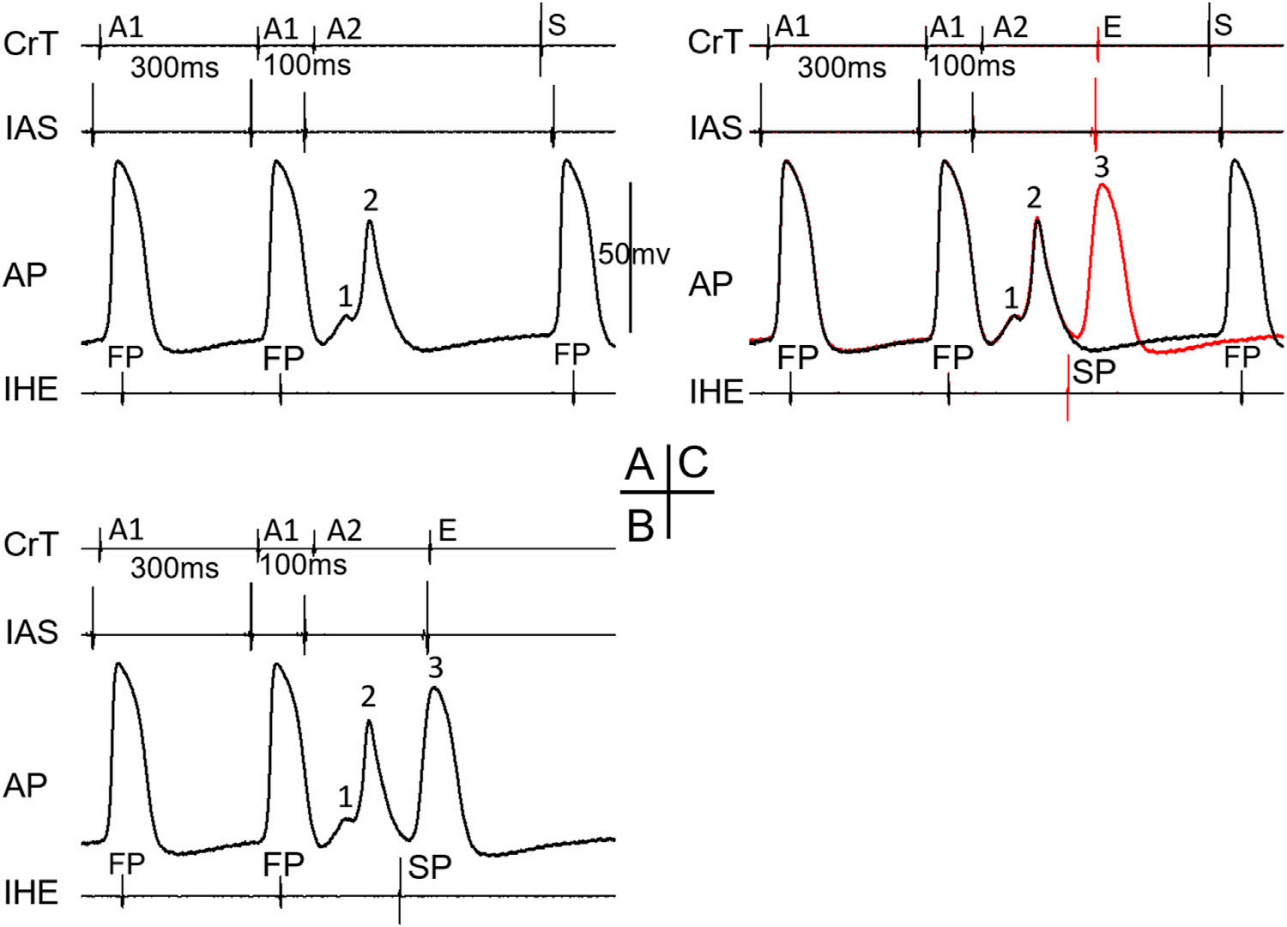
Example of the formation of intranodal/nodal-atrial reentry that likely resulted in His bundle activation. The recordings were taken at the same A1A2 = 100 ms. The A2 beat resulted in an AV block in **(A)**. The AP recording showed an initial very small local response caused by the FP wave front (1) and a second larger local response by the SP wave front (2). In another episode with the same A1A2, however, AV conduction occurred **(B)**. The AP recording showed identical initial local FP and SP responses (1, 2) but was followed by an additional activation (3), which was associated with an atrial echo beat (E). The overlap of electrical signals in **(A,B)** is shown in **(C)**. E, atrial echo beat; S, sinus beat.

Figure 5 shows an example that His bundle activation was made possible by the formation of intranodal/nodal–atrial reentry and its subsequent conduction; otherwise, an AV block would occur. At A1A2 = 100 ms, in this case, the AP first showed a very small local response (A, 1), indicating a “dying” FP wave front, and then a later, larger local response (A, 2), indicating a retrograde SP wave front, which is associated with an AV block (A). However, at another episode with the same A1A2 (B), the initial responses of the cell were identical to the blocked beat in A, showing the same local responses as the failing FP and SP wave fronts. Thus, it is likely that the premature beat was not conducted to the His bundle. However, in this case, a reentry (intranodal/nodal–atrial) occurred, which is evidenced by the additional (3rd) AP and the atrial echo beat. This was associated with His bundle activation. In this case, most likely, the formation of an intranodal/nodal–atrial reentry was subsequently conducted to the His bundle, rather than a direct conduction by the premature beat. It should be pointed out that if the AH interval were measured in this case, it would be an artificial/misinterpreted time/event, or pseudo-interval, because the AH interval did not really reflect the fact that the premature beat failed to conduct to the His bundle, and only the subsequent reentry was conducted to the His bundle.

### A jump in the AV conduction curve is always associated with the formation of intranodal/nodal–atrial reentry

**FIGURE 6:**
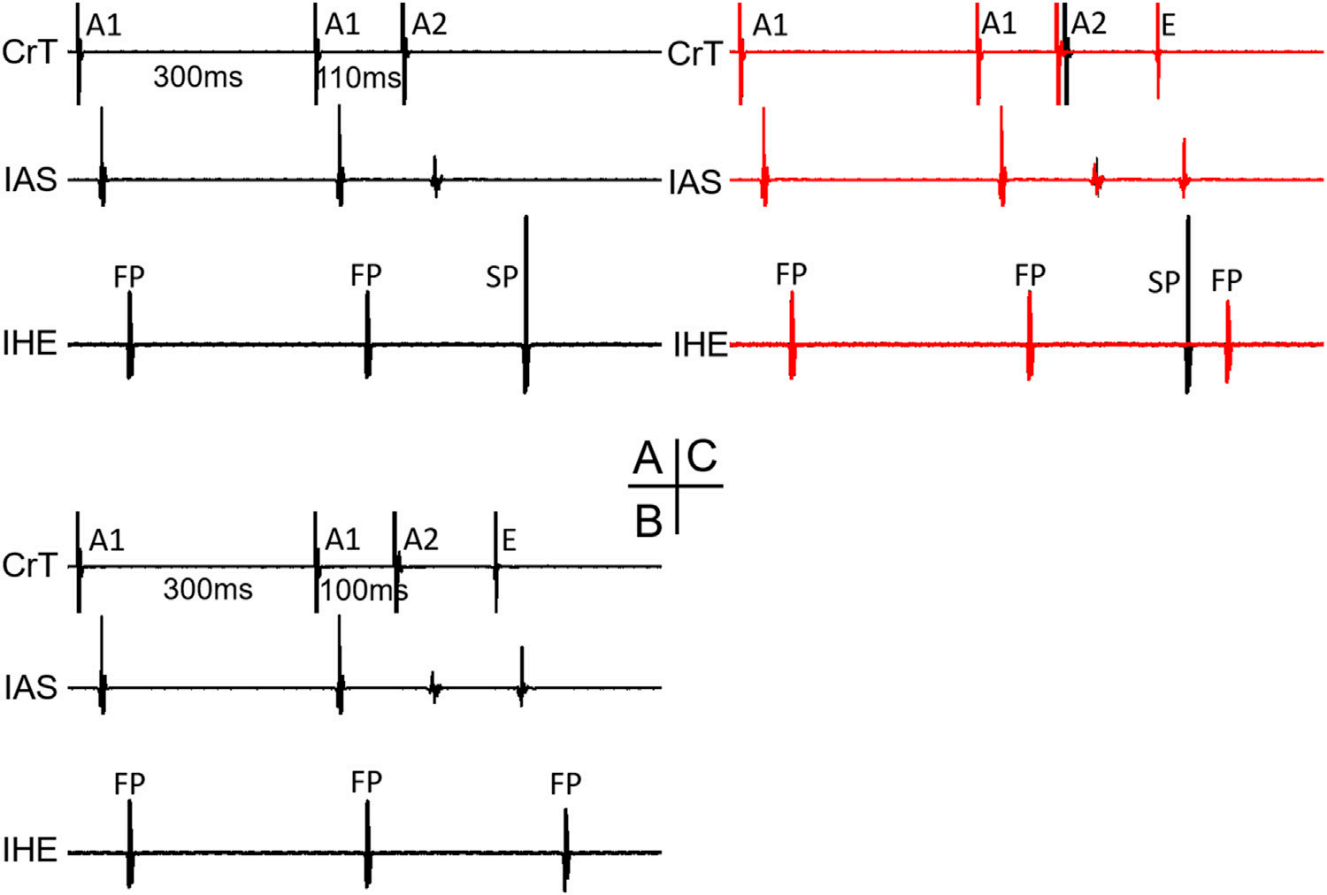
Example of a jump that resulted in a His electrogram with an FP pattern. At the premature beat preceding the jump [A1A2 = 110 ms, **(A)**], the IHE showed SP conduction. At the beat resulting in a jump (A1A2 = 100 ms), the IHE showed an FP pattern **(B)** with a much longer AV delay (AH interval increased by 62 ms compared with the previous beat). The overlapped signals of the two premature beats are shown in **(C)**. Abbreviations are the same as in Figure 3.

Figure 6 shows an example of a jump that resulted in an HE with an SP–FP pattern observed in the 7/11 preparations. As already stated, the beat before the jump was conducted by the SP (Figure 6A). At the beat resulting in a jump (Figure 6B), the His bundle activation was suddenly further delayed and showed an FP pattern (low IHE) from SP conduction (high IHE) in the previous beat (Figure 6A). The jump was associated with an atrial echo beat, and the His bundle activation occurred much later than the atrial echo activation, indicating that the reentry circuit was likely located in the upper part of the node or involved nodal–atrial tissue. The overlapped signals of the two beats are shown in Figure 6C. Given that the FP had previously failed before the jump, the reappearance of FP conduction at an even shorter prematurity cannot be attributed to direct conduction by the premature beat along the typical FP. Consequently, we postulated that the jump was most likely a result of reentry formation and subsequent conduction. Qualitatively, this happened in all 7/11 preparations, showing a jump resulted in a HE with an SP–FP pattern.

**FIGURE 7:**
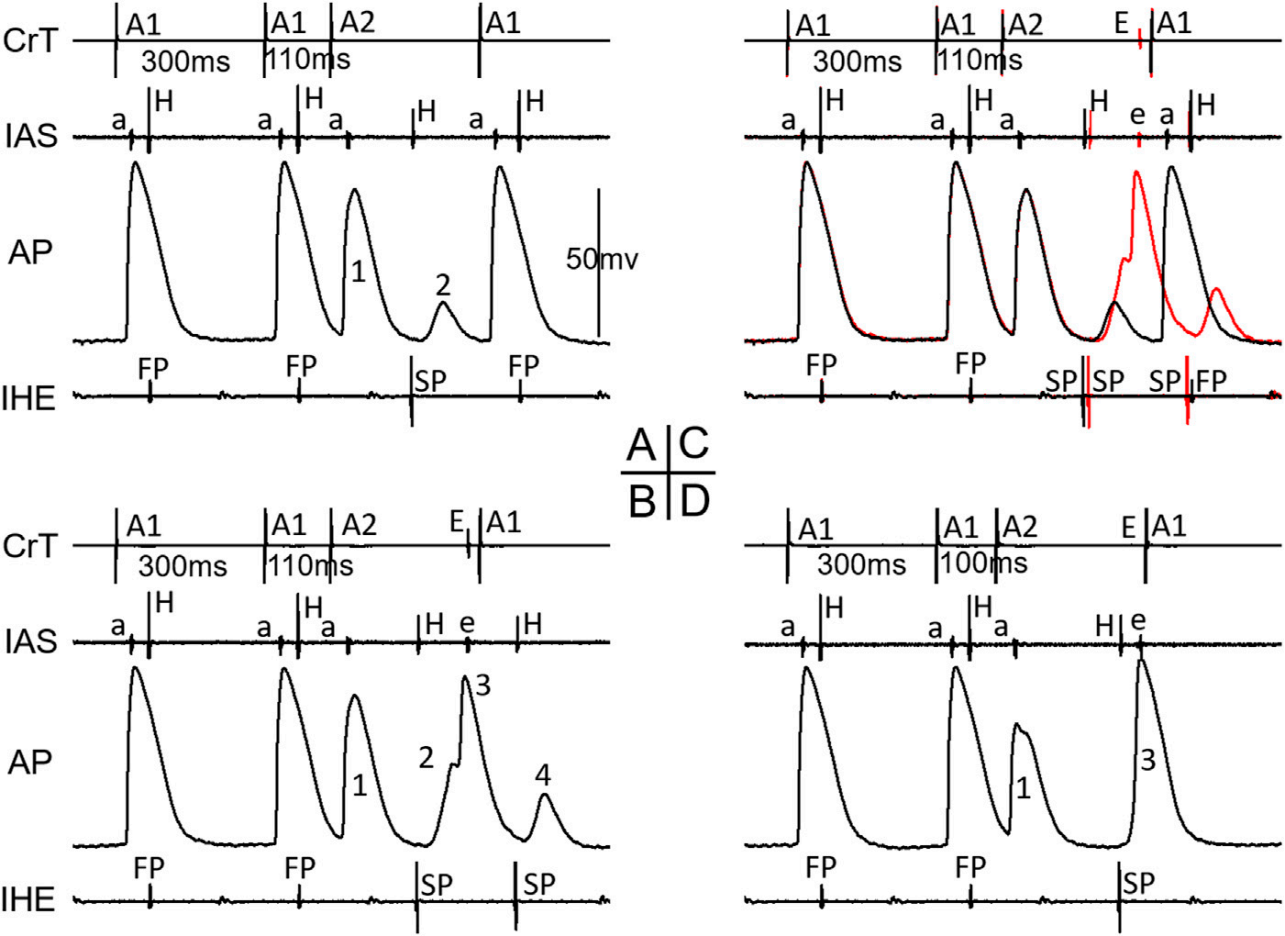
Example of a jump resulted in a His electrogram with an SP-pattern. At the premature beat preceding the jump (A1A2 = 110 ms, **(A)**, IHE showed SP conduction. The AP showed a decremental FP wave-front (1) and a small SP wave-front (2). However, in another episode with the same A1A2 = 110 ms **(B)**, there were additional wave-fronts (3, 4) besides the FP and SP wave-fronts, indicating complex reentry already formed. The overlap of A and B is shown in **(C)**, clearly indicating these events. At the beat resulting in a jump **(D)**, the AP showed further decreased FP wave-front (1), disappearance of the SP wave-front, and a suddenly delayed HE, associated with additional AP (3) and atrial echo beat. Please see detailed explanation in the text. Note that in this case, IAS recorded both atrial electrogram (a) and superior His electrogram (H). Also note that the last A1 stimulation (in B and D) was delivered shortly after the atrial echo beat and was not able to capture the atria (no atrial activation in IAS). Abbreviations are the same as in Figure 3.

Figure 7 shows an example of a jump that resulted in a HE with an SP–SP pattern in the 4/11 preparations. Again, the beat before the jump was already conducted by the SP (A, A1A2 = 110 ms). In this case, the AP was recorded from a cell located in the superior nodal domain. The AP showed a decremental FP wave front (1) and a small SP wave front (2) at the beat preceding the jump (A). However, in another episode with the same A1A2, there were additional wave fronts (3, 4) besides the FP and SP wave fronts, indicating complex reentry had already formed (B). This was also supported by the atrial echo beat and the additional HE with an SP pattern. The overlap of A and B is shown in C, clearly indicating these events. At the beat resulting in a jump (D), the AP showed the following: further decreased FP wave front (1), disappearance of the SP wave front, and a suddenly delayed HE, associated with the additional AP (3) and atrial echo beat. The delayed HE was most likely due to the formation of reentry and its subsequent conduction. Similar reentrant events occurred in other preparations, displaying a jump resulted in a HE with an SP–SP pattern.

**FIGURE 8:**
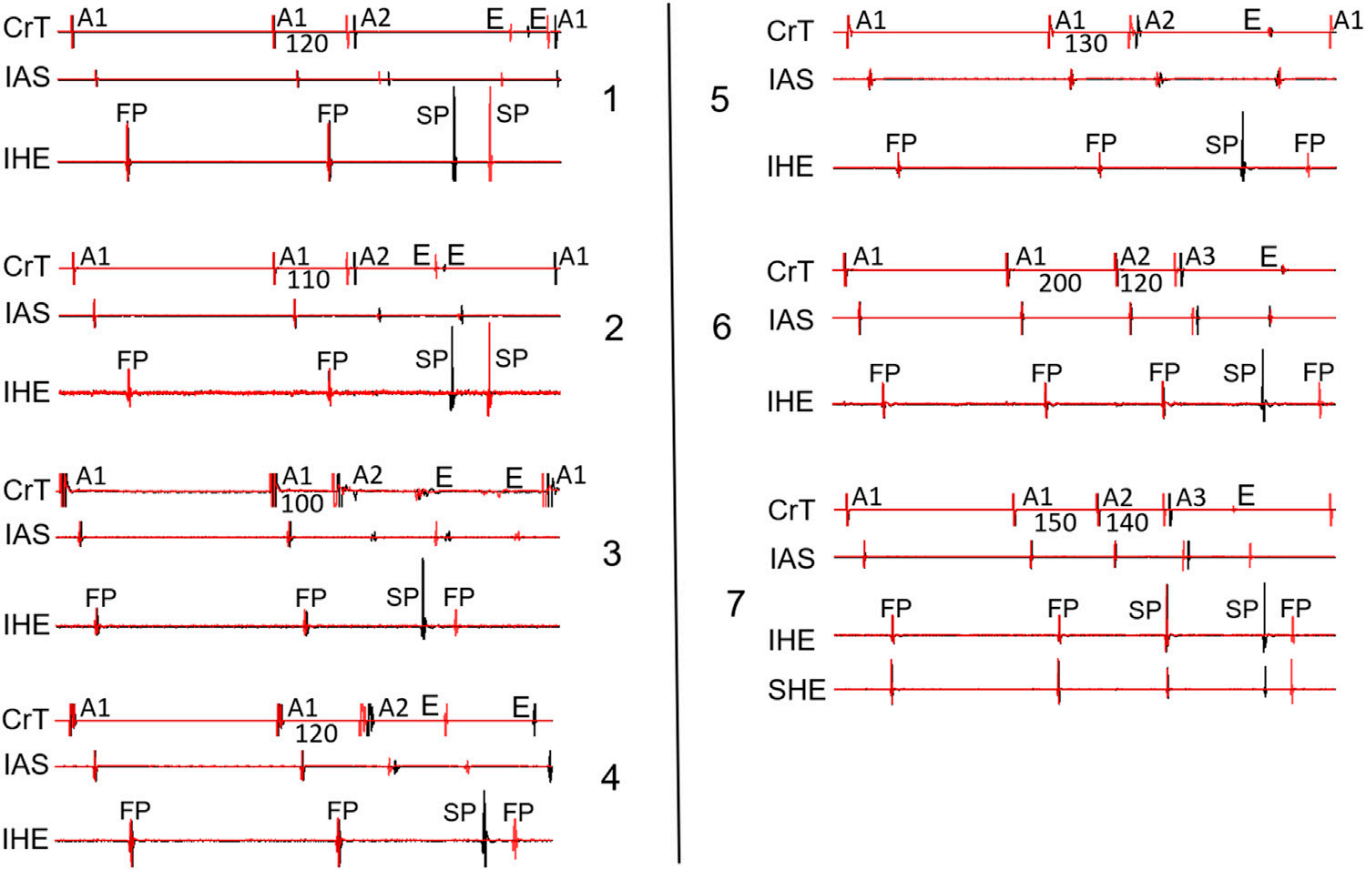
Overlapped signals showing the beat just before the jump (black) and the beat with a jump (red) from the remaining seven preparations not shown in the previous figures. Two cases (panels 1–2) showed an SP–SP pattern, and 5 cases (panels 3–7) showed an SP–FP pattern. Note that in 2 cases (panels 6–7), the jump was revealed by the A1A2A3 pacing protocol. SHE: superior His electrogram. All other abbreviations are the same as in Figure 2.

Figure 8 shows the recordings in the remaining seven preparations with a jump (which have not been illustrated in previous figures). Note that all jumps were associated with reentry formation, evidenced by the atrial echo beat(s).

## Discussion

### Major findings

In this study, we investigated whether a transition from the FP to SP is the cause of the jump in the AV conduction curve by using a novel index of dual-pathway AV nodal conduction (HE alternans) and intracellular action potential recording from fibers in the AV node. We have demonstrated that the transition from FP to SP conduction was smooth (without a jump) and a jump in the AV conduction curve always occurred after the transition from FP to SP conduction. Thus, a jump in the AV conduction curve is not caused by a switch from FP to SP conduction as assumed and accepted for almost half a century. Our results indicate that a jump in the AV conduction curve is always associated with the formation of intranodal/nodal–atrial reentry. Furthermore, the His bundle activation of the jumped beat shows either an SP–FP pattern or an SP–SP pattern. Taken together, it is highly likely, albeit not definitive, that a jump in the AV conduction curve is caused by the formation of reentry and its subsequent conduction to the His bundle, rather than a simple switch from FP to SP conduction.

### AV nodal dual-pathway electrophysiology and the jump in the AV conduction curve

**FIGURE 9:**
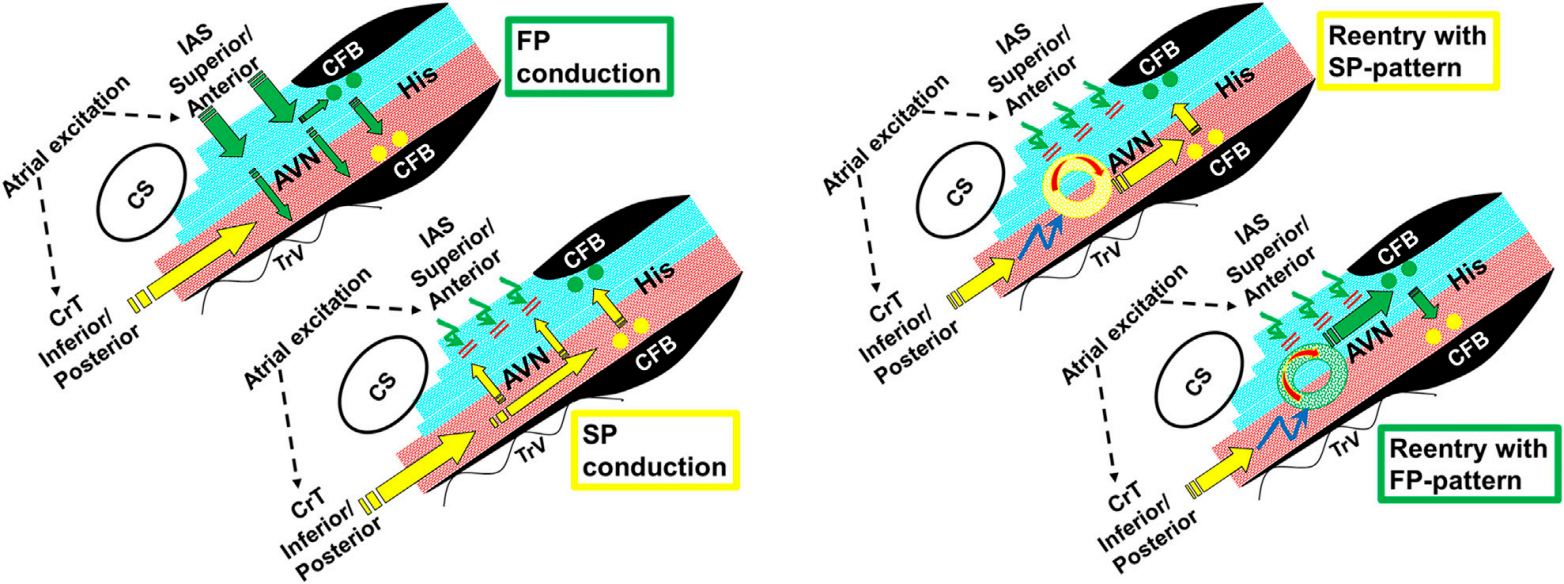
Schematic illustrations showing FP and SP conduction (left panel) and the formation of reentry and its subsequent conduction to the His bundle, resulting in an SP or FP pattern (right panel). The electrical excitation sequence and functional dissociation (superior nodal–His bundle domain in blue, and inferior nodal–His bundle domain in red) are shown during dual-pathway conduction (left panel). The FP wave front conducts in a superior-to-inferior direction across fiber orientation within the AV node (green arrows) and results in a superior input into the superior His bundle domain (green dots). At short A1A2, the FP fails to propagate in a “superior-to-inferior direction” (curved green arrows). This permits the SP to conduct along the AV nodal fiber orientation in the inferior nodal domain (yellow arrows), resulting in an inferior input into the inferior His bundle domain (yellow dots) (Zhang, 2014). The right panel shows that at even shorter A1A2, the FP fails to propagate and the SP may also fail to directly conduct to the His bundle (curved blue arrows). However, the SP propagation may initiate an intranodal/nodal–atrial reentry (circles), and the reentrant beat could subsequently conduct to the superior His bundle (FP pattern) or inferior His bundle (SP pattern). AVN, AV node; His, His bundle; CFB, central fibrous body; CS, coronary sinus; TrV, tricuspid valve; IAS, interatrial septum; CrT, crista terminalis.

The AV nodal dual-pathway electrophysiology was initially developed as the underlying mechanism of AVNRT, the pathology underlying the most common type of PSVT (Zhang, 2016; Gonzalez et al., 2020). Based on their experimental data, Moe et al. (Moe et al., 1956; Mendez and Moe, 1966) were the first to propose that the upper part of the AV node could be functionally and spatially dissociated into β- and α-pathways, and the two pathways communicate with a final common pathway before the His bundle. It was found that the β-pathway had a longer ERP but a shorter AV delay, whereas the α-pathway had a shorter ERP but a longer AV delay. Denes et al. (1973) were the first to report the presence of AV nodal dual pathways in patients. They named the pathway producing the shorter AV delay as the FP (assuming it conducts fast) and the pathway with the longer AV delay as the SP (assuming it conducts slow). Note that this assumption may be incorrect because the FP and SP utilize different routes in the AV node (Zhang, 2016). We demonstrated previously that the FP conduction is from the interatrial septum to the anterior/superior nodal domain and superior His bundle domain, whereas the SP conduction is from the crista terminalis to the posterior/inferior nodal domain and inferior His bundle domain (Zhang, 2014). This concept is also shown in Figure 9 (left panel). A detailed discussion of the potential FP and SP routes was given by Zhang (2016). A recent report suggests that the AV node (part of the primary ring) initially communicates with the inferior atrial wall before the perpendicular atrial septal–nodal connection developed (Hikspoors et al., 2022). Further research is needed to determine if this unique embryonic development plays a role in dual-pathway electrophysiology.

The dual-pathway model has provided a convenient explanation for AVNRT and also provided a clinical criterion for diagnosing AV nodal dual-pathway electrophysiology (Zhang, 2016; Gonzalez et al., 2020). It is assumed that the FP is responsible for AV conduction at a longer cycle length. Once the cycle length shortens to reach the ERP of the FP, a conduction block occurs in the FP. Electrical propagation now has to go through the SP, which results in a much longer AV conduction time. Thus, a switch from FP to SP conduction would result in a jump in the AV conduction time. Accordingly, a jump in the AV conduction curve has been conveniently assumed as the hallmark and the clinical criterion for dual-pathway electrophysiology (Mazgalev et al., 2001; Zhang, 2016; Gonzalez et al., 2020) despite the fact that this assumption has never been confirmed.

Contrary to the current assumption, this study has provided the first evidence that a jump in the AV conduction curve is not an indicator of the transition from FP to SP conduction. In fact, the transition from FP to SP conduction occurred consistently before the jump and was typically smooth (without a jump). We found that the jumps always occurred after the transition from FP to SP conduction, near the end of the AV conduction curve (shorter A1A2 region on the left side of the curve). Interestingly, for the beats that resulted in a jump, an SP–FP pattern was observed in 7/11 preparations and an SP–SP pattern was observed in the remaining 4 preparations. In addition, our results showed that the jumps were always associated with the formation of intranodal/nodal–atrial reentry. This finding is consistent with early clinical reports that the jumps were associated with atrial echo beats (Denes et al., 1973). Thus, our data suggested that the jumps were most likely caused by the formation of intranodal/nodal–atrial reentry and the subsequent conduction of the reentrant beat, rather than a simple switch from FP to SP conduction by the premature beat. This concept is shown in Figure 9 (right panel). In fact, the premature beat most likely failed to conduct directly to the His bundle. As such, a jump measured from the atrial premature beat to the His bundle activation is an artificial, misinterpreted AV conduction time.

In this study we have also provided evidence that intranodal reentry could occur without atrial involvement (Figure 3) or only with partial atrial activation (Figure 4). However, in many cases, intranodal activations were associated with atrial echo beats. When there was 1:1 nodal–atrial activation, it was difficult to insist that these reentries were intranodal, not involving the atrial tissue.

### Study limitations

As stated, this was a retrospective study based on experimental records from previous experiments. In most preparations, the AV nodal conduction curve was taken with an A1A2 pacing protocol. An A1A2A3 pacing protocol was utilized only in a dozen preparations from which multiple conduction curves were available. We noticed that in two of the preparations, a jump was revealed by A1A2A3 pacing but not by A1A2 pacing. Thus, the incidence of a jump could be higher if the A1A2A3 pacing protocol were consistently applied, as reported in patients (Kuo et al., 1999).

In this study, we applied the clinical criterion to define the jump as there is no specific criterion in rabbits. However, the conduction time in rabbit AV is generally shorter than that in humans (AV conduction time is typically in the range of 60–90 ms in rabbits (Zhang and Mazgalev, 2012) *versus* 120–200 ms in humans). A 50-ms jump used in patients might be too high for rabbits. If a lower standard of 30 ms or 40 ms were used, then there would be more cases with a jump.

As demonstrated, intracellular AP recording from the AV nodal fibers could provide valuable information about detecting intranodal reentries and tachycardia, which otherwise might not be observed on atrial or His electrograms (concealed intranodal reentries). These complex intranodal/nodal–atrial reentries could help elucidate the potential mechanism underlying the jump. Nevertheless, the value of AP recording is highly dependent on the fiber location from where the AP is recorded. For example, only APs from fibers near the reentrant circuit could reflect the reentrant wave fronts. In addition, intracellular AP recording was a time-consuming, tedious procedure and could only be obtained from a limited number of fibers. It was impractical or impossible to map out the entire reentrant circuit with AP recordings. Moreover, it was also very difficult to record the same or similar APs with a similar response from different preparations due to variations in anatomy and electrophysiology in different preparations. Nevertheless, with AP recordings, we have shown the existence of pure intranodal reentry (Figures 3, 4) and provided evidence that the formation of intranodal/nodal–atrial reentry and its subsequent conduction was likely responsible for the jump in the AV nodal conduction curve.

Although there are anatomical similarities in the AV node in rabbits and humans, interspecies differences exist (Tawara, 1906; Macias et al., 2022). Thus, further electrophysiological evidence is needed to confirm these findings in humans.

## Conclusion

A jump in the AV nodal conduction curve is not produced by the transition from FP to SP conduction, as it has been assumed. Our data suggest that the formation of intranodal or nodal-atrial reentry by the premature beat and the subsequent conduction of the reentrant beat to the His bundle is likely the cause of the jump.

## Data Availability

The original contributions presented in the study are included in the article/Supplementary Material; further inquiries can be directed to the corresponding author.
